# Mr. Marshall's Case of Concussion of the Brain

**Published:** 1813-06

**Authors:** Samuel Marshall

**Affiliations:** Member of the Royal College of Surgeons in London. Leeds


*To the Editors of the Medical and Physical Journal.*

GENTLEMEN,

HAVING just now seen an article from Juvenis, in your valuable publication for February last, on the subject of Concussion of the Brain, I have to request you will, if you think proper, present him in your next with the following case and observations; which, if not a direct answer to his query, will, I hope, throw some little light on this important subject, and tend to confirm him in the opinion he has, perhaps, already adopted.

E. K., a Dutch seaman on-board the H. C. S. Coutts, while lying in the river Tigris, on a late India voyage, by some misfortune lost his balance while at work on the main yard, and fell down upon deck. Happening to see the poor fellow fall as I sat at dinner in the cuddy, I ran to him immediately, and finding his breathing stertorous and his pulse oppressed, with but little sign of life, considering it a severe case of concussion, I directly opened a vein in the arm, and, by the time I had taken somewhat less than a pound, the pulse rose, the breathing became easier, and every circumstance appeared more promising. This, however, was a very temporary delusion, as the sequel will show. I had now time to examine more particularly the nature and extent of the injury. On a very careful inspection of the head, not the least external mark of injury was to be found there; but the left arm, in addition to a dislocation of the os humeri, had sustained a dreadful compound fracture of the lower part of that bone, being literally pounded to atoms; the internal condyle, on which he seems first to have pitched, being buried a full inch deep, even in a hard knot in the plank, and requiring the carpenter with his chissel and mallet to extricate it. This member, being so completely shattered, I did not long hesitate in determining to amputate. The dislocation, however, came first to be reduced; but this, from the man's weak state, did not require much effort. I now had him very carefully conveyed to his birth on the gun deck, and went for my amputating instruments; but, on my return, had the mortification to find his countenance ghastly, his pulse sunk, with a cold clammy sweat, and every sign of approaching death; which event, in spite of wine, volatile alcali, and other cordials, did take place about an hour after the accident.

I have to regret not having been allowed to open the body, which might possibly have afforded some explanation of this unfortunate case; but, upon reflection afterwards, it struck me very forcibly as being one of those cases of which a very respectable author of the present day observes, "Now the surgeon bleeds, but the landlady gives a dram, and I think that in this case the landlady is right."

The inference I drew from this case was, that by waiting a few minutes, or by the judicious administration of stimuli, the vis vitæ might be so far restored as to bear up under the vigorous method of treatment required in these severe accidents.

Should the recital of this catastrophe prove a beacon to any other sea-faring surgeon in similar circumstances, I should deem it the most profitable act of my life.

I am, Gentlemen,
Your obedient Servant,
SAMUEL MARSHALL,
Member of the Royal College of Surgeons in London.

*Leeds, May* 8, 1813.

